# Type of Delivery, Neuropsychological Development and Intelligence in Twin Births

**DOI:** 10.3389/fpsyg.2019.00972

**Published:** 2019-05-03

**Authors:** María-José González-Valenzuela, Ernesto González-Mesa, Olga Cazorla-Granados, Dolores López-Montiel

**Affiliations:** ^1^Facultad de Psicología, Universidad de Málaga, Málaga, Spain; ^2^Facultad de Medicina, Universidad de Málaga, Málaga, Spain; ^3^Colegio Santa Rosa de Lima, Málaga, Spain

**Keywords:** type of delivery, neuropsychological development, general intelligence, retrospective cohort study, twin births

## Abstract

Based on a retrospective cohort design with 6-year-old children born in twin births, the relationship between verbal, non-verbal, global neuropsychological development, general intelligence and type of delivery has been studied. To this end, the possible effect of third gestational, obstetric and neonatal variables, such as maternal age at delivery, fetal presentation, gestational age, newborn weight and Apgar at minute one, was controlled. The exposed cohort includes children born by cesarean section, and the unexposed cohort is composed of children born vaginally with or without induction. A total of 124 children were evaluated in their 1st year of primary school using the Child Neuropsychological Maturity Questionnaire, Kaufman’s Intelligence Test and the medical histories of the children collected after birth. By means of binary logistic regression analysis, it has been found that the type of delivery is presented as an independent risk factor for disorders in verbal, non-verbal and global development and for the general intellectual difficulties of children born of multiple births. These results suggest the need to analyze in future prospective studies with broader samples the relationship between different types of obstetric and perinatal variables of birth type and infant neuropsychological development and general intelligence, in order to prevent possible psychological alterations from birth.

## Introduction

Certain obstetric conditions frequently associated with multiple pregnancies, such as maternal age, preterm birth, birth weight perinatal hypoxia, or labor complications, have been implicated in developmental delays among children (Calame et al., 2004; Jansson-Verkasalo et al., 2004; David and Dean, 2007; Lollar and Cordero, 2007; Wolke et al., 2008; Cattani et al., 2010; Dall’oglio et al., 2010; Mercier et al., 2010; Kurth and Haussmann, 2011; Bidzan and Bieleninik, 2013; González-Valenzuela et al., 2015a,b). Preterm birth and low birth weight are important determinants of psychological development, since it has been shown that the earlier the birth in terms of weeks of gestation and the lower the birth weight, the greater the delay observed in psychological development, particularly in the first few years (Jansson-Verkasalo et al., 2004; Mercier et al., 2010; Aarnoudse-Moens et al., 2012; Zwicker and Harris, 2012; González-Valenzuela et al., 2015b). Maternal age under 18 and over 40 is also a cause of high-risk pregnancy and can affect the mother’s health and the baby’s development (David and Dean, 2007; Lollar and Cordero, 2007; Kurth and Haussmann, 2011; Bidzan and Bieleninik, 2013).

However, there are relatively few studies that relate type of delivery to psychological problems. Furthermore, there appears to be no consensus with regard to consideration of risk caused by cesarean birth compared to vaginal delivery in the newborn and the mother (Hogle et al., 2003; Villar et al., 2007; Guerra et al., 2011). Some studies establish that cesarean birth carries a risk for the baby and the mother, whereas others do not state the existence of any differences with regard to vaginal delivery. The type of delivery is, therefore, for many authors a topic that is still open to discussion regarding its relationship with the development of children born in single and multiple deliveries. For some, short- and long-term infant outcomes are affected by the mode of delivery (Matthew and Neena, 2012; Karlström et al., 2013). It is well known that respiratory problems during the early neonatal period increase two–threefold after an elective cesarean birth (van den Berg et al., 2001). The instances of hypoglycemia (Hägnevik et al., 1984), low temperature (Christensson et al., 1993), delayed breastfeeding, difficulties in maternal bonding with the newborn and in neurodevelopment also increased (Carlander et al., 2010; Asztalos et al., 2016; Polidano et al., 2017). Many studies have suggested that a cesarean section affects long-term offspring outcomes regarding metabolic syndrome, the immune system, dentition, malignancies and nervous system development, describing plausible biological mechanisms although still failing to prove casualty (Pasupathy and Smith, 2008; Hyde et al., 2012). On the other hand, others argue that a lower risk of asphyxia, encephalopathy, and intracranial hemorrhage was found with cesarean sections compared to vaginal delivery (Hogle et al., 2003; Villar et al., 2007).

The mode of delivery has been directly related to biochemical and structural changes in the central nervous system, the consequences of which are not well known. Thus, recent studies reveal the existence of *in vitro* biochemical brain changes related to the active work of a normal childbirth, noting some deterioration in the functional development of the hippocampus, which leads to the existence of lifelong neuropsychological dysfunctions in cases in which there was no labor due to a scheduled cesarean section (Simón-Areces et al., 2012).

The rate of multiple pregnancies has recently increased, largely due to the use of assisted reproduction techniques and the increase in maternal age at the time of birth (Mesa and Peral, 2011). These pregnancies lead to an overload for the mother and often result in preterm and low-birth weight infants as well as posing added challenges arising from the need to address ‘simultaneously’ the second stage of labor for two fetuses. The second infant is more vulnerable due to complications such as cord prolapse, detachment of the placenta, dystocia due to cervical spasms, or trauma from intrauterine manipulation in cases of fetal extraction due to non-cephalic presentation. This happens especially in cases of great discordance of fetal weight or extremely low fetal weight, although not when gestational age is <34 weeks in both (Asztalos et al., 2016; Girsen et al., 2016).

However, although there are international recommendations for multiple childbirth assistance, the evidence is weak and is usually based on expert opinions and some retrospective studies (MacKay et al., 2006). In fact, whereas there are studies that show an increased risk of morbidity in a vaginal birth in the second twin compared to the first twin (Wen et al., 2004a,b; Armson et al., 2006; Yang et al., 2006; González-Mesa et al., 2016), others fail to demonstrate in the second twin the short- or long-term benefits of elective cesarean-sections compared to vaginal delivery (Rabinovici et al., 1987; Greig et al., 1992; Hogle et al., 2003; Asztalos et al., 2016; Girsen et al., 2016).

In this context, the main objective of this study is to estimate the relationship between verbal, non-verbal and global neuropsychological development, general intelligence, and the type of delivery in children born from twin births by the age of six. To this end, other potentially confusing gestational, obstetric and neonatal variables were controlled: maternal age at delivery, fetal presentation, gestational age, weight of the newborn and Apgar at minute one.

## Materials and Methods

### Design

An epidemiological study of retrospective cohorts was designed. The risk factor (type of delivery) preceded the result (verbal development, non-verbal development, global development, and general intelligence at 6 years of age). The cohort exposed children born to twin births by cesarean section (exposed to cesarean section) and the cohort did not expose children born to twin births by vaginal delivery (not exposed to cesarean section), from the selected random sample of all twin births at the Hospital Materno-Infantil of Málaga during the year 2005.

### Participants

**FIGURE 1:**
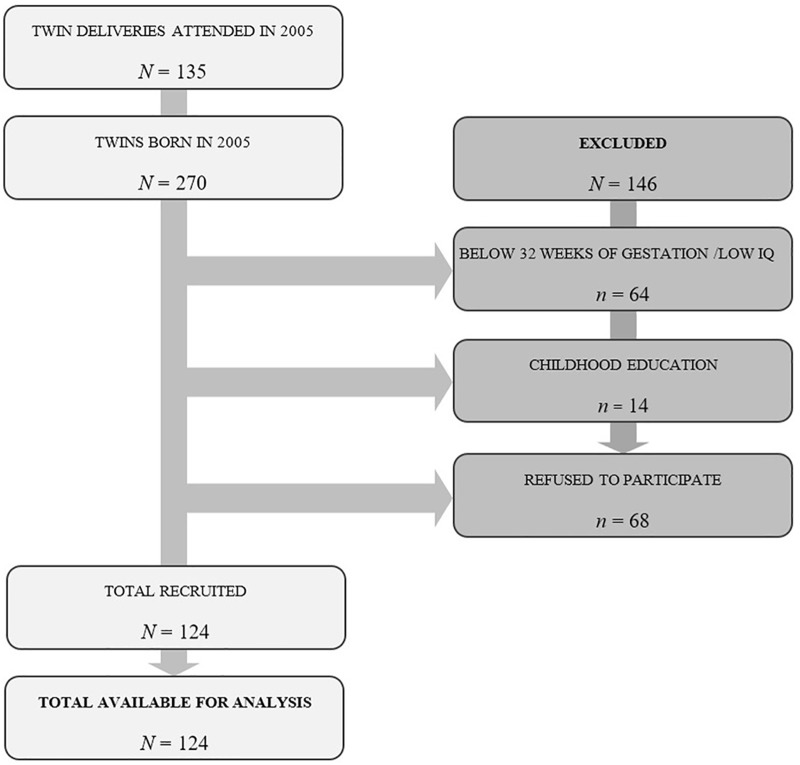
Flow diagram of participants through each stage of the study.

The study population comprised children who were born in the Materno-Infantil Hospital in Málaga in 2005, were at least 6 years of age, had started their compulsory education (1st year of primary education), and showed no signs of possible disorders in their psychological development that could be irrevocably established. The hospital is a tertiary center in the Spanish national health system, in which 7,120 children were born in 2005, of which 270 children were born in twin births. Of these twins, 64 children born at less than 32 weeks of gestation, and 14 children who were studying a lower school level were excluded from the study. Sixty-eight children could not be recruited because their mothers could not be located due to a change of residence or they did not want to participate in the study when they were located (Figure 1). Therefore, the inclusion criteria applied to participants in the study were as follows: they had to be 6 years of age, be in Year 1 of Primary Education, have been born after 32 weeks of gestation, and the mothers had to be locatable and be willing to participate in the research.

Thus, the selected population is made up of 270 children born in twin births, of whom 124 children could be assessed, corresponding to 62 twin births ranging from 74 to 86 months old (*M* = 79.42, *SD* = 3.44), of whom 62 are male (50%) and 62 are female (50%). In this sample, 51 mothers (41.1%) and 58 fathers (46.8%) had a primary level of education (primary and pre-secondary studies); 38 mothers (30.6%) and 40 fathers (32.3%) had an intermediate level of education (junior high and high school, technical, and non-technical); and 35 mothers (28.2%) and 26 fathers (21%) had a higher education (college and graduate). The maternal age at the time of delivery ranged from 22 to 45 years of age (*M* = 33.2, *SD* = 4.27); the gestational age of the infants was between 32 and 41 weeks (*M* = 35.14, *SD* = 2.07); the weight of the infants was between 1179 and 3080 g (*M* = 2137.76, *SD* = 432.79), fetal presentation was cephalic in 80 deliveries (64.5%) and non-cephalic (breech and transverse) in 44 (35.5%), and the score on the Apgar 1 test ranged between 4 and 10 points (*M* = 8.41, *SD* = 1.18). Of the total sample, 84 (67.7%) were born vaginally and 40 (32.3%) were born by Cesarean section. The choice of cesarean section was indicated in 17 births (42.5%) due to maternal problems (mother’s pathologies and/or non-progression in delivery) and 23 (57.5%) due to problems of fetal origin (malposition of the first twin and/or the loss of fetal wellbeing).

### Instruments

To assess neuropsychological development, we used the “Cuestionario de Madurez Neuropsicológica Infantil” (CUMANIN [Child Neuropsychological Maturity Questionnaire]; Portellano et al., 2009). This questionnaire is intended to assess the degree of neuropsychological maturity reached by the subject in different areas of development and the possible presence of brain dysfunction (Portellano et al., 2009). It consists of a standardized battery of tests that assess Verbal Development (VD), Non-Verbal Development (NVD), and Global Development (GD) of children aged between 3 and 6 years. Various tests are included in the Appendix. The total VD and NVD score is the sum of the scores obtained in each sub-test (number of correct answers). The tests that assess GD are the same as those used for the evaluation of VD and NVD, and the total score is the sum of the scores obtained in all of the tests (total number of correct answers). Cronbach’s alpha coefficient values in all the scales ranged between 0.57 and 0.92, and in these ages, the range was between 0.83 and 0.88. The correlations between the indices of difficulty and discrimination of the items of the classical theory of the tests and the corresponding estimators of these parameters of the response to the item were high. The children were considered to have a verbal, non-verbal or global development disorder when they scored below the 25th percentile, respectively, in accordance with the evaluation criteria established for the test (Portellano et al., 2009). In other words, variables relating to neuropsychological development were defined as follows: presence of a developmental disorder if the child scored less than the 25th percentile: absence of a developmental disorder if the score was equal to or higher than the 25th percentile.

To measure general intelligence (GI), we used the Kaufman Brief Intelligence Test -K-BIT- (Kaufman and Kaufman, 2000), which assesses verbal and non-verbal intelligence at ages ranging from 4 to 90 years. It consists of two subtests: Vocabulary and Matrices. The *Vocabulary* test evaluates verbal ability related with school learning (crystallized thinking) and has two parts, Expressive Vocabulary and Definitions, which measure knowledge of words and the formation of concepts. The Matrices test assesses non-verbal skills and the ability to solve new reasoning problems through figurative and abstract visual stimuli (fluid thinking), based on the subject’s ability to perceive relationships and complete analogies between objects. The total Intelligence score is the sum of the scores obtained in each of the subtests (total number of correct answers). The reliability coefficients of the scales ranged from 0.80 to 0.90. Children were considered to be at risk of presenting general intellectual difficulties when they scored below the 25th percentile, according to the evaluation criteria established for the test (Kaufman and Kaufman, 2000). In other words, the variable intelligence was defined as follows: risk of intellectual difficulties if the child scored less than the 25th percentile: no risk of intellectual difficulties if the score was equal to or higher than the 25th percentile. The rest of the variables considered in the study were assessed through the medical histories of the mothers and their children. The independent variable was the type of delivery, treated dichotomously, with the categories being vaginal delivery (induced and non-induced) and Cesarean section. Considering that the main objective of this study is to examine the effect of the type of delivery on twins’ neuropsychological development and intelligence in the presence of third variables that can produce confounding and interaction phenomena (treated as control variables), we evaluated the following dichotomized gestational, obstetric, and neonatal variables (maternal age, fetal presentation, gestational age and weight of the newborn and Apgar 1). Maternal age represented the age of the mother at the time of delivery; gestational age indicated the number of weeks’ gestation of the baby when born; the newborn’s birth weight was expressed in grams; cephalic presentation was classified as either cephalic or non-cephalic (breech and transverse); and the Apgar-1 test measured the baby’s heart rate, muscle tone and other signs to determine whether they need additional or emergency medical assistance within a range of 10 points. These variables were transformed into a categorical dichotomic scale as per González-Mesa et al. (2016), in accordance with clinical criteria. Maternal age at the time of delivery was classified as being over or under 35 years of age; fetal presentation was cephalic or non-cephalic (breech or transverse); gestational age of the newborn, with categories above or below 37 weeks, and above or below 34 weeks; the weight of the newborn was considered either above or below 1,500 g; and Apgar at 1 min, with categories of above or below 7 points.

### Procedure

This study was carried out in accordance with the recommendations and approval of the Research Ethics Committee (Comité de Ética de la Investigación) of the Hospital Regional Universitario Carlos Haya in Málaga. All mothers of the subjects gave written informed consent in accordance with the Declaration of Helsinki.

After obtaining the corresponding authorization from the ethics committee, we obtained the necessary data to contact the mothers by phone. During these telephone calls, which lasted around 10 min, we explained the objectives and development of the research, and proposed an appointment for the psychological assessment of the child. This evaluation was carried out in the consultation rooms of the Hospital Materno-Infantil in Málaga. The mothers signed the informed consent form at the beginning of the assessment session, and, subsequently, the evaluators were left alone with the child in order to carry out the assessment.

The session began with the individual administration of the “Cuestionario de Madurez Neuropsicológica Infantil” [Child Neuropsychological Maturity Questionnaire] and, subsequently, the Kaufman Intelligence Test. The tests were administered by three experienced psychologists, and the estimated administration time was 30 and 15 min, respectively.

Finally, some of the authors of the study gathered data on the obstetric and perinatal variables of the selected cases of mothers who agreed to participate in the study through a review of clinical records in the hospital and using the identification number of the selected mothers after identifying them from among all the records of the mothers who gave birth in 2005.

### Statistical Analysis

In accordance with the objective and design of this study, multivariate regression analysis was chosen as the main statistical technique. When selecting the most appropriate kind of regression in accordance with the properties of the data, an assessment was conducted *a priori* of the parametric assumptions required of linear regression models (linearity, normality, and homoscedasticity) by means of the analysis of scatter graphs and histograms, and the Kolmogorov–Smirnov test of normality for all variables, considering statistical significance to be proven if the probability associated with the statistic was higher than 0.05 (two-tailed). Having observed its non-fulfillment for almost all the variables, and taking into account the nature of the main independent variable studied here, binary multivariate logistic regression was chosen. In order to apply this technique correctly, all the variables that were originally quantitative, both the independent and the control variables, were dichotomized in order to improve the efficiency of the analysis and clarity of the interpretation, in accordance with the criteria specified previously.

Before estimating the regression models, the differences between the originally quantitative variables, according to type of delivery, were explored, as well as the bivariate relationship between all the variables studied. These preliminary analyses were conducted with a view to evaluating the main relationships studied, detecting possible masking variables and selecting the most appropriate ones for the regression models. For the analysis of differences, the Mann–Whitney *U* and Student’s *t*-test were applied, as applicable. For the analysis of relationships, contingency tables and Pearson’s χ^2^ independence tests were applied, considered statistically significant if the probability associated with these statistics was less than 0.05 (two-tailed). To quantify the strength of the association of the effect of cesarean birth on verbal, non-verbal, global and general intelligence development, as well as on each control variable, the unadjusted or crude Odds Ratio (*OR*) was estimated without adjusting for the confounding variables and their corresponding 95% confidence intervals (95% *CI*). *OR*s whose intervals did not include the null value (*OR* = 1) were considered statistically significant.

Finally, binary logistic regressions with each dependent variable of the study (verbal and non-verbal development, global development, and general intelligence) were used to evaluate the possible interaction (modification of the main effect studied) between the control variables and the independent variable type of delivery, as well as the possible confusion between the control variables and the main relationship evaluated (the effect of the type of delivery on verbal and non-verbal development, overall development and general intelligence), if statistically possible. In this case, we chose the control variables that, in the bivariate analyses, had more than 10% of cases in each cell, a probability associated with a Pearson’s χ^2^ statistic of less than 0.05 in the independence tests, and an *OR* whose interval of 95% confidence was statistically significant.

For the construction of the regression models for each dependent variable, the recommendations of Hosmer and Lemeshow (2000) and Kleinbaum and Klein (2001) were followed. It was based on a maximum hierarchical model, in which the statistically significant interactions and the variables implied in them would be conserved, whenever possible. Having eliminated the non-significant interactions sequentially from the model, according to the corresponding test of statistical significance, the possible confounding factors were studied, considering the possible bias on the regression coefficients, the precision (amplitude) of their intervals of confidence and their standard error, as well as non-statistical criteria, such as the change in the magnitude of the *OR*. Hence, confusion would be detected when the magnitude of the *OR*, which evaluates the strength of association between the independent and the dependent variable, changed clinically in an important way (10% between the gross and adjusted association measures) when eliminating a variable from the equation, with respect to the initial model. The variables retained would eventually be included in the construction of the most appropriate model. The goodness of fit of the selected regression models was evaluated using the Likelihood Ratio test and the Hosmer–Lemeshow test, and the Wald Chi-square test for the statistical significance of the regression coefficients. To assess the validity of the models overall, the Nagelkerke adjusted determination coefficient was used. The regression models were estimated manually by the researcher, based on the results obtained in each step. Statistical analysis was carried out using the Statistical Package for the Social Sciences (SPSS), version 23.

## Results

Following the descriptive-exploratory analysis of the data, comparisons were made between the means and the means of the ranges of originally quantitative dependent variables (verbal development, non-verbal development, global development, and general intelligence) and the control variables (maternal age, gestational age, weight of newborn, and Apgar-1), as a function of the independent variable ‘type of delivery’ (vaginal/cesarean). Statistically significant differences were found for verbal development, non-verbal development and global development, as well as general intelligence between the mean and the mean ranges obtained by the 84 children born by vaginal delivery and those of the 40 born by cesarean section.

And no statistically significant differences were found for maternal age, gestational age, or weight of the newborn, or for Apgar 1, between the mean ranges of the children born by vaginal delivery and those born by cesarean section (see Table 1).

**Table 1:** Descriptive and exploratory analysis of originally quantitative variables and statistical tests for means and mean ranks differences.

Variables							Type of delivery										
			
	*N* = 124						Vaginal delivery *n* = 84				Cesarean *n* = 40				Statistical test		
				
	*M*	*SD*	*Range*	*K–S*	*df*	*p*	*M*	*SD*	*Range*	*MR*	*M*	*SD*	*Range*	*MR*	*U*	*Z*	*p*
VD	21.18	3.70	10-27	0.15	124	0.000^*^	21.70	3.38	10-27	67.01	20.08	4.13	10-26	53.04	1301.50	-2.04	0.042
NVD	42.94	5.67	23-55	0.13	124	0.000^*^	43.65	5.56	23-52	68.52	41.45	5.77	29-55	49.85	1174.00	-2.71	0.007
GD	64.12	7.84	39-79	0.11	124	0.000^*^	65.36	7.46	39-76	69.09	61.53	8.08	45-79	48.66	1126.50	-2.96	0.003
															***t***	***df***	
GI	48.17	8.92	21-72	0.05	124	0.200	49.50	7.79	29-70	-	45.38	10.48	21-72	-	2.46	122	0.015
WNB	2137.76	432.79	1179-3080	0.05	124	0.200	2154.48	445.26	1310-3080	-	2102.65	408.59	1179-2905	-	0.62	122	0.535
															***U***	***Z***	
MA	33.24	4.27	22-45	0.09	124	0.006^*^	32.71	4.03	22-40	59.21	34.35	4.42	28-45	69.40	1404.00	-1.48	0.139
GA	35.14	2.08	32-41	0.08	124	0.020^*^	35.09	1.99	32-40	62.31	35.25	2.27	32-41	62.90	1664.00	-0.08	0.932
A1	8.41	1.18	4-10	0.43	124	0.000^*^	8.36	1.23	4-10	61.11	8.53	1.06	5-9	63.85	1586.00	-0.51	0.612


*VD, verbal development; NVD, non-verbal development; GD, global development; GI, general intelligence; WNB, weight of newborn (grams); MA, maternal age (years); GA, gestational age (weeks); A1 (Apgar 1); M, mean; SD, standard deviation; K–S, Kolmogorov–Smirnov test; MR, mean rank; U, U-Mann–Whitney test; t, t-Student test. ^∗^The distribution is not normally distributed at 0.05 level.*

In summary, statistically significant differences were found in all dependent variables according to the type of delivery, with higher scores in children born vaginally. On the contrary, no statistically significant differences were found in the quantitative control variables according to the type of delivery.

The analysis of the bivariate relationships between the independent variable type of delivery (vaginal/cesarean) and the control variables potentially masking the effect (maternal age, fetal presentation, gestational age, weight of the newborn, and Apgar 1), to detect interactions, is summarized in Table 2. There is a statistically significant relationship between type of delivery and fetal presentation [χ^2^(2,124) = 45.53, *p* < 0.001].

**Table 2:** Bivariate associations between independent variable (type of delivery) and the control variables (gestational, obstetric, and neonatal variables).

Control variables	Categories	Total	Independent variable Type of delivery		*^∗^*χ^2^	*p*
					
		*N* = 124	Vaginal delivery *n* = 84 (67.7%)	Cesarean *n* = 40 (32.3%)		
Maternal age (years)	Under 35	88	64 (72.7%)	24 (27.3%)	3.44^a^	0.063
	Over 35	36	20 (55.6%)	16 (44.4%)		
Gestational age of newborn (weeks)	Over 37	40	29 (72.5%)	11 (27.5%)	0.61^a^	0.434
	Under 37	84	55 (65.5%)	29 (34.5%)		
Gestational age of newborn (weeks)	Over 34	90	60 (66.7%)	30 (33.3%)	0.17^a^	0.677
	Under 34	34	24 (70.6%)	10 (29.4%)		
Fetal presentation	Cephalic	80	71 (88.8%)	9 (11.3%)	45.53^a^	0.000
	Non-cephalic	44	13 (29.5%)	31 (70.5%)		
Weight of newborn (grams)	Over 1500	112	76 (67.9%)	36 (32.1%)	0.07^b^	0.999
	Under 1500	12	8 (66.7%)	4 (33.3%)		
Apgar 1	Over 7	113	76 (57.3%)	37 (32.7%)	0.13^b^	0.755
	Under 7	11	8 (72.7%)	3 (27.3%)		


*^∗^Pearson χ^*2*^. ^*a*^0% of cells with an expected frequency less than 5. ^*b*^25% of cells with an expected frequency less than 5*.

Tables 3–6 summarize the analyses of the bivariate relationships between the independent variable type of delivery (vaginal birth/cesarean section) and the dependent variables VD, NVD, GD, and GI (presence/absence of disorder) and the potentially effect-masking control variables, respectively. The frequency distributions for each category of the independent variable in relation to the categories of the other variables, along with the significance of the Pearson χ^2^ statistic and the *OR* and its corresponding confidence interval, are presented.

**Table 3:** Bivariate associations between verbal development, type of delivery and the control variables.

Variables	Categories	Total	Dependent variable Verbal development		^∗^χ^2^	*p*	*OR*	95% *CI*	
							
		*N* = 124	No VD delay*n* = 92 (74.2%)	VD delay*n* = 32 (25.8%)				Lower	Upper
**Independent**									
Type of delivery	Vaginal	84 (67.7%)	68 (81%)	16 (19%)	6.21	0.013	2.83	1.23	6.52
	Cesarean	40 (32.3%)	24 (60%)	16 (40%)					
**Control**									
Maternal age (years)	Under 35	88 (71%)	68 (77.3%)	20 (22.7%)	1.50^a^	0.221	1.70	0.72	3.99
	Over 35	36 (29%)	24 (66.7%)	12 (33.3%)					
Gestational age of newborn (weeks)	Over 37	40 (32.3%)	30 (75%)	10 (25%)	0.02^a^	0.887	1.06	0.44	2.53
	Under 37	84 (67.7%)	62 (73.8%)	22 (26.2%)					
Gestational age of newborn (weeks)	Over 34	90 (72.6%)	65 (72.2%)	25 (27.8%)	0.66^a^	0.414	0.67	0.26	1.74
	Under 34	34 (27.4%)	27 (79.4%)	7 (20.6%)					
Fetal presentation	Cephalic	80 (64.5%)	64 (80%)	16 (20%)	3.97^a^	0.046	2.28	1.00	3.97
	Non-cephalic	44 (35.5%)	29 (65.9%)	16 (36.4%)					
Weight of newborn (grams)	Over 1500	112 (90.3%)	83 (71.1%)	29 (25.9%)	0.05^b^	0.999	0.95	0.24	3.76
	Under 1500	12 (9.7%)	9 (75%)	3 (25%)					
Apgar 1	Over 7	113 (91.1%)	84 (74.3%)	29 (25.7%)	0.01^b^	0.999	0.92	0.23	3.70
	Under 7	11 (8.9%)	8 (72.3%)	3 (27.3%)					


*VD, verbal development; OR, odds ratio; CI, confidence interval. ^∗^Pearson χ^*2*^. ^*a*^0% of cells with an expected frequency less than 5. ^*b*^25% of cells with an expected frequency less than 5.*

**Table 4:** Bivariate associations between non-verbal development, type of delivery and the control variables.

Variables	Categories	Total *N* = 124	Dependent variable		Non-verbal development	*^∗^*χ^2^	*p*	*OR*	95% *CI*	
							
			No NVD delay *n* = 93 (75%)	NVD delay *n* = 31 (31%)				Lower	Upper
**Independent**									
Type of delivery	Vaginal	84 (67.7%)	68 (81%)	16 (19%)	4.92	0.027	2.55	1.10	5.90
	Cesarean	40 (32.3%)	25 (62.5%)	15 (48.4%)					
**Control**									
Maternal age (years)	Under 35	88 (71%)	65 (73.9%)	23 (26.1%)	0.21^a^	0.648	0.81	0.32	2.02
	Over 35	36 (29%)	28 (77.8%)	8 (22.2%)					
Gestational age of newborn (weeks)	Over 37	40 (32.3%)	28 (70%)	12 (30%)	0.78^a^	0.375	0.68	0.29	1.59
	Under 37	84 (67.7%)	65 (77.4%)	19 (22.6%)					
Gestational age of newborn (weeks)	Over 34	90 (72.6%)	65 (72.2%)	25 (27.8%)	1.35^a^	0.245	0.55	0.20	1.50
	Under 34	34 (27.4%)	28 (82.4%)	6 (17.6%)					
Fetal presentation	Cephalic	80 (64.5%)	64 (80%)	16 (20%)	3.00^a^	0.083	2.07	0.90	4.74
	Non-cephalic	44 (35.5%)	28 (63.6%)	15 (34.1%)					
Weight of newborn (grams)	Over 1500	112 (90.3%)	85 (75.9%)	27 (24.1%)	0.49^b^	0.729	1.57	0.43	5.63
	Under 1500	12 (9.7%)	8 (66.7%)	4 (33.3%)					
Apgar 1	Over 7	113 (91.1%)	86 (76.1%)	27 (23.9%)	0.83^b^	0.465	0.55	0.15	2.02
	Under 7	11 (8.9%)	7 (63.3%)	4 (36.4%)					


*NVD, non-verbal development; OR, odds ratio; CI, confidence interval. ^∗^Pearson χ^*2*^. ^*a*^0% of cells with an expected frequency less than 5. ^*b*^25% of cells with an expected frequency less than 5*.

**Table 5:** Bivariate associations between global development, type of delivery and the control variables.

Variables	Categories	Total *N* = 124	Dependent variable Global development		^∗^χ^2^	*p*	*OR*	95% *CI*	
							
			No GD delay *n* = 93 (75%)	GD delay *n* = 31 (25%)				Lower	Upper
**Independent**									
Type of delivery	Vaginal	84 (67.7%)	70 (83.3%)	14 (21%)	9.64	0.002	3.69	1.58	8.64
	Cesarean	40 (32.3%)	23 (57.5%)	17 (42.5%)					
**Control**									
Maternal age (years)	Under 35	88 (71%)	65 (73.9%)	23 (26.1%)	0.21^a^	0.648	0.81	0.32	2.02
	Over 35	36 (29%)	28 (77.8%)	8 (22.2%)					
Gestational age of newborn (weeks)	Over 37	40 (32.3%)	30 (75%)	10 (25%)	0.00^a^	0.999	1.00	0.42	2.38
	Under 37	84 (67.7%)	63 (75%)	21 (25%)					
Gestational age of newborn (weeks)	Over 34	90 (72.6%)	67 (74.4%)	23 (25.6%)	0.05^a^	0.816	0.89	0.35	2.25
	Under 34	34 (27.4%)	26 (76.5%)	8 (23.5%)					
Fetal presentation	Cephalic	80 (64.5%)	67 (83.8%)	13(16.3%)	9.20^a^	0.002	3.57	1.53	8.30
	Non-cephalic	44 (35.5%)	26 (59.1%)	18 (40.9%)					
Weight of newborn (grams)	Over 1500	112 (90.3%)	86 (76.8%)	26 (23.2%)	1.96^b^	0.291	2.36	0.69	8.07
	Under 1500	12 (9.7%)	7 (58.3%)	5 (41.7%)					
Apgar 1	Over 7	113 (91.1%)	85 (75.2%)	28 (24.8%)	0.03^b^	0.999	0.88	0.22	3.54
	Under 7	11 (8.9%)	8 (72.3%)	3 (27.3%)					


*GD, global development; OR, odds ratio; CI, confidence interval. ^∗^Pearson χ^*2*^. ^*a*^0% of cells with an expected frequency less than 5. ^*b*^25% of cells with an expected frequency less than 5*.

**Table 6:** Bivariate associations between general intelligence, type of delivery and the control variables.

Variables	Categories	Total *N* = 124	Dependent variable General intelligence		^∗^χ^2^	*p*	*OR*	95% *CI*	
							
			No GI delay *n* = 91 (73.4%)	GI delay *n* = 33 (26.6%)				Lower	Upper
**Independent**									
Type of delivery	Vaginal	84 (67.7%)	68 (81%)	16 (19%)	5.42	0.020	2.62	1.15	6.00
	Cesarean	40 (32.3%)	24 (60%)	16 (40%)					
**Control**									
Maternal age (years)	Under 35	88 (71%)	68 (77.3%)	20 (22.7%)	2.34^a^	0.126	1.92	0.82	4.46
	Over 35	36 (29%)	23 (63.9%)	13 (36.1%)					
Gestational age of newborn (weeks)	Over 37	40 (32.3%)	32 (80%)	8 (20%)	1.32^a^	0.250	1.69	0.68	4.19
	Under 37	84 (67.7%)	59 (70.2%)	25 (29.8%)					
Gestational age of newborn (weeks)	Over 34	90 (72.6%)	64 (71.1%)	26 (28.9%)	0.87^a^	0.351	0.63	0.24	1.64
	Under 34	34 (27.4%)	27 (79.4%)	7 (20.6%)					
Fetal presentation	Cephalic	80 (64.5%)	61 (76.3%)	19 (23.8%)	0.94^a^	0.331	1.49	0.66	3.39
	Non-cephalic	44 (35.5%)	30 (68.2%)	14 (31.8%)					
Weight of newborn (grams)	Over 1500	112 (90.3%)	83 (74.1%)	29 (25.9%)	0.31^b^	0.731	1.43	0.40	5.11
	Under 1500	12 (9.7%)	8 (66.7%)	4 (33.3%)					
Apgar 1	Over 7	113 (91.1%)	81 (71.7%)	32 (28.3%)	1.89^b^	0.285	3.95	0.48	32.13
	Under 7	11 (8.9%)	10 (90.9%)	1 (9.1%)					


*GI, general intelligence; OR, odds ratio; CI, confidence interval. ^∗^Pearson χ^*2*^. ^*a*^0% of cells with an expected frequency less than 5. ^*b*^25% of cells with an expected frequency less than 5*.

In general, of the total sample selected, there were no alterations in VD, NVD, GD, and GI, in 92 (74.2%), 93 (75%), 93 (75%), and 91 (73.4%) children, respectively. Alterations in VD, NVD, GD, and GI were detected in 32 (25.8%), 31 (25%), 31 (25%), and 33 (26.6%) children, respectively (see Tables 3–6).

Analyses between the criterion variable VD, the independent variable (type of delivery) and the control variables were found to be related [χ^2^(2, *N* = 124) = 6.21, *p* < 0.05]. Out of the 40 (32.3%) children who were born by cesarean section, 16 (40%) did not pass the VD scale, whereas out of the 84 (67.7%) children who were born vaginally, 16 (19%) did not pass the VD scale. The crude *OR* indicates that cesarean section multiplied the probability of presenting alterations in VD by 2.83, *OR* = 2.83, 95% *CI* [1.23, 6.52] (see Table 3). Fetal presentation and VD were also significantly related [χ^2^(2, *N* = 124) = 3.97, *p* < 0.05]. Out of the 44 (35.5%) children with non-cephalic presentation (breech or transverse), 16 (36.4%) did not pass the VD test, and out of the 80 (64.5%) with cephalic presentation, 16 (20%) did not pass the VD test. The crude *OR* indicates that fetal presentation multiplied the probability of presenting alterations in VD by 2.28, *OR* = 2.28, 95% *CI* [1.00, 3.97]. We found no statistical significance between VD and the other control variables evaluated (see Table 3).

The variables NVD and type of delivery were also related [χ^2^(2, *N* = 124) = 4.92, *p* < 0.05]. Out of the 40 (32.3%) children born by cesarean section, 15 (48.4%) did not pass the NVD scale, whereas out of the 84 (67.7%) children who were born vaginally, 16 (19%) did not pass the NVD scale. It appears that birth by cesarean section nearly triples the likelihood of presenting alterations in NVD, *OR* = 2.55, 95% *CI* [1.10, 5.90] (see Table 4). Again, we found no statistical significance between NVD and the other control variables considered (see Table 4).

A positive relationship was also found between GD and type of delivery [χ^2^(2, *N* = 124) = 9.64, *p* < 0.01]. Out of the 40 (32.3%) children born by cesarean section, 17 (42.5%) did not pass the GD scale, whereas out of the 84 (67.7%) who were born vaginally, 14 (21%) did not pass it. Cesarean delivery appears to triple the likelihood of presenting alterations in GD, *OR* = 3.69, 95% *CI* [1.58, 5.90] (see Table 4). GD and fetal presentation were also related [χ^2^(2, *N* = 124) = 9.20, *p* < 0.01]. Out of the 44 (35.5%) children with non-cephalic presentation (breech or transverse), 18 (40.9%) did not pass the GD test, and out of the 80 (64.5%) with cephalic presentation, 13 (16.3%) did not pass the GD test. The associated *OR* indicates that fetal presentation multiplies the probability of presenting alterations in GD by 3.57, *OR* = 3.57, 95% *CI* [1.53, 8.30] (see Table 5). We found no statistical significance between GD and the other control variables evaluated (see Table 5).

The dependent variable GI and the independent variable type of delivery were also related [χ^2^(2, *N* = 124) = 5.42, *p* < 0.05]. Out of the 40 (32.3%) children born by cesarean section, 16 (40%) did not pass the GI scale, and out of the 84 (67.7%) who were born vaginally, 16 (19%) did not pass this scale. Again, we observed that cesarean delivery almost triples the probability of risk of difficulties in GI, *OR* = 2.62, 95% *CI* [1.15, 6.00] (see Table 6). We found no statistical significance between GI and the rest of the control variables (see Table 6).

Subsequently, binary logistic regressions were performed for each of the study’s dependent variables [verbal development, non-verbal development, global development, and general intelligence (see Tables 7, 8)], which included the clinically plausible control variables that had more than 10% of cases in each cell in the prior bivariate analyses, a probability associated with a Pearson χ^2^ statistic lower than 0.05 in the tests of independence and an *OR* with a statistically significant 95% *CI.*

**Table 7:** Multivariate logistic regression analysis for verbal and non-verbal development, adjusted by potential interaction and confounding factors.

*Variables*		*b*	*SE*	Wald χ^2^	*df*	*p*	*OR*	95% *CI*	
								
								Lower	Upper
**VD**									
Model 1	**Type of delivery**^(a)^	0.80	0.77	1.08	1	0.298	2.23	0.49	10.10
	Fetal presentation^(b)^	0.29	0.72	0.16	1	0.688	1.33	0.32	5.55
	Type of delivery × Fetal presentation	0.07	1.07	0.00	1	0.944	1.07	0.13	8.91
	Constant	-1.49	0.36	23.75	1	0.000	0.22		

	^∗^χ^2^(3, *N* = 124) = 6.34, *p* = 0.096								

Model 2	**Type of delivery**^(a)^	0.84	0.53	2.46	1	0.116	2.32	0.81	6.62
	Fetal presentation^(b)^	0.32	0.53	0.37	1	0.541	1.38	0.48	3.94
	Constant	-1.50	0.29	25.99	1	0.000	0.22		

	^∗^χ^2^(2, *N* = 124) = 6.34, *p* = 0.042; χ*^2^*(2, *N* = 124) = 0.05, *p* = 0.997; *R*^2^ = 0.07								

Model 3	**Type of delivery**^(a)^	1.04	0.42	5.98	1	0.014	2.83	1.23	6.52
	Constant	-1.44	0.27	27.11	1	0.000	0.23		

	^∗^χ^2^(1, *N* = 124) = 5.97, *p* = 0.015; *R*^2^ = 0.07								

NVD									
Model 1	**Type of delivery**^(a)^	0.93	0.43	4.76	1	0.029	2.55	1.10	5.90
	Constant	-1.44	0.27	27.11	1	0.000	0.25		

	^∗^χ*^2^*(1, *N* = 124) = 4.73, *p* = 0.030; *R*^2^ = 0.05								


*VD, verbal development; NVD, non-verbal development; OR, odds ratio; CI, confidence interval. Variables reference categories: (a) = Vaginal delivery; (b) = Cephalic. ^∗^Goodness-of-fit tests for logistic regression models: Global test χ^*2*^; Hosmer–Lemeshow χ^*2*^; Nagelkerke R^*2*^*.

**Table 8:** Multivariate logistic regression analysis for global development and general intelligence, adjusted by potential interaction and confounding factors.

Variables		*b*	*SE*	Wald χ^2^	*df*	*p*	*OR*	95% *CI*	
								
								Lower	Upper
**GD**									
Model 1	**Type of delivery**^(a)^	1.11	0.78	2.01	1	0.156	3.05	0.65	14.21
	Fetal presentation^(b)^	0.99	0.69	2.08	1	0.149	2.71	0.70	10.50
	Type of delivery × Fetal presentation	-0.49	1.05	0.22	1	0.636	0.61	0.07	4.78
	Constant	-1.80	0.34	28.09	1	0.000	0.16		

	^∗^χ^2^(3, *N* = 124) = 11.54, *p* = 0.009; χ^2^(2, *N* = 146) = 0.00, *p* = 0.999; *R*^2^ = 0.13								

Model 2	**Type of delivery**^(a)^	0.83	0.53	2.42	1	0.120	2.31	0.80	6.62
	Fetal presentation^(b)^	0.78	0.53	2.14	1	0.143	2.19	0.76	6.25
	Constant	-1.75	0.32	30.43	1	0.000	0.17		

	^∗^χ^2^(2, *N* = 124) = 11.32, *p* = 0.003; χ*^2^*(2, *N* = 124) = 0.22, *p* = 0.894; *R*^2^ = 0.13								

**Model 3**	**Type of delivery**^(a)^	1.31	0.43	9.08	1	0.003	3.69	1.58	8.64
	Constant	-1.61	0.29	30.22	1	0.000	0.20		

	^∗^χ^2^(1, *N* = 124) = 9.21, *p* = 0.002; *R*^2^ = 0.10								

**GI**									
**Model 1**	**Type of delivery**^(a)^	0.96	0.42	5.24	1	0.022	2.62	1.14	6.00
	Constant	-1.37	0.27	25.50	1	0.000	0.25		

	^∗^χ^2^(1, *N* = 124) = 5.22, *p* = 0.022; *R*^2^ = 0.06								


*GD, global development; GI, general intelligence; OR, odds ratio; CI, confidence interval. Variables reference categories: (a) = Vaginal delivery; (b) = Cephalic. ^∗^Goodness-of-fit tests for logistic regression models: Global test χ^*2*^; Hosmer–Lemeshow χ^*2*^; Nagelkerke R^*2*^.*

First, the main relationship studied between VD and type of delivery was adjusted by fetal presentation and the interaction between type of delivery and fetal presentation. Fetal presentation was considered a potential modifier of the main effect under study as well as a potential confounding variable because it was statistically associated with both the independent variable (see Table 2) and the dependent variable (see Table 3). Following adjustment by VD and type of delivery and eliminating the interaction term [Wald χ^2^(1, *N* = 124) = 0.00, *p* = 0.944] and the variable fetal presentation [Wald χ^2^(1, *N* = 124) = 0.37, *p* = 0.541] in two successive steps, the third estimated model was statistically significant [χ^2^(1, *N* = 124) = 5.97, *p* < 0.05], and the independent variable type of delivery was the only significant variable [Wald χ^2^(1, *N* = 124) = 5.98, *p* < 0.05], with an *OR* = 2.83, 95% CI [1.23, 6.52]. Therefore, cesarean birth is a risk factor for VD delay, such that the *OR* obtained for the variable type of delivery indicates that the risk of VD delay is 2.83 times more likely among infants born by cesarean section than those born vaginally. Regarding the explanatory capacity of this model, 7% of the variability in the response variable is explained by the estimated logistic regression model (Nagelkerke’s *R*^2^ = 0.07) (see Table 7).

In addition to the main relationship between the dependent variable NVD and the independent variable type of delivery, no significant relationship was found between any of the control variables in the previous analyses (see Table 4). Following adjustment by type of delivery, the logistic model was significant [χ^2^(1, *N* = 124) = 4.73, *p* < 0.05], and therefore the independent variable was also significant [Wald χ^2^(1, *N* = 124) = 4.76, *p* < 0.05] with an *OR* = 2.55, 95% *CI* [1.10, 5.90]. According to the estimated model, it was observed that cesarean delivery is also a risk factor for NVD delay. In addition, the *OR* obtained indicated that the risk of NVD delay is 2.55 times more likely among infants born by cesarean section than among those born vaginally. Regarding the explanatory capacity of the model, 5% of the variance of the NVD variable is explained by the estimated model (Nagelkerke’s *R*^2^ = 0.05) (see Table 7).

As with VD, the main relationship studied between GD and type of delivery was adjusted by fetal presentation and interaction between type of delivery and fetal presentation because, in this case, fetal presentation was statistically related both to the independent variable (see Table 2) and the dependent variable (see Table 5). After eliminating the interaction term [Wald χ^2^(1, *N* = 124) = 0.22, *p* = 0.636] and the variable fetal presentation [Wald χ^2^(1, *N* = 124) = 2.14, *p* = 0.143] in two successive steps, the third estimated model was significant [χ^2^(1, *N* = 124) = 9.21, *p* < 0.01], and only the independent variable type of delivery was significant [Wald χ^2^(1, *N* = 124) = 9.08, *p* < 0.01], with an *OR* = 3.69, 95% *CI* [1.58, 8.64]. Therefore, cesarean birth is a risk factor for GD delay, such that the *OR* obtained for the variable type of delivery indicated that the risk of NVD delay was 3.69 times more likely among infants born through cesarean section than those born vaginally. 10% of the variance for the variable non-verbal development is explained by the estimated model (Nagelkerke’s *R*^2^ = 0.10) (see Table 8).

Regarding the dependent variable GI, no significant relationship was found between type of delivery and the control variables in the previous analyses performed (see Table 6), in addition to the main relationship studied. Therefore, GI was adjusted by type of delivery, generating a significant estimated binary logistic model [χ^2^(1, *N* = 124) = 5.22, *p* < 0.05], and therefore, the independent variable type of delivery as well [Wald χ^2^(1, *N* = 124) = 5.24, *p* < 0.05] with an *OR* = 2.62, 95% *CI* [1.14, 6.00]. Therefore, birth by cesarean section was again a risk factor for developing GI difficulties, such that the *OR* obtained for the variable type of delivery indicated that the risk of GI difficulties was 2.62 times more likely among infants born through cesarean section than those born vaginally. In relation to the variance of the response variable explained by the model, 6% of the variance found for the variable GI is explained by the variable included in the estimated model (Nagelkerke’s *R*^2^ = 0.06) (see Table 8).

## Discussion

The objective of this study was to obtain an unbiased estimate of the relationship between neuropsychological development and general intelligence on the one hand, and type of delivery of the other.

The results confirm these relationships and indicate that cesarean delivery in twin births is a possible risk factor, independent of the other factors considered, for the presence of disorders in VD, NVD, and GD and for difficulties in the children’s GI. None of the control variables considered modified the effect of type of delivery on neuropsychological development and general intelligence. Specifically, the risk of having a neuropsychological developmental disorder and intellectual difficulties at 6 years of age is about three times more likely among newborns in twin births through cesarean section than those born vaginally. However, the discrete explanatory capacity of the estimated models must be considered, which agrees with the final inclusion of a single independent variable.

The results, therefore, are in line with studies that establish that the type of delivery involves risks for babies, and that, in particular, cesarean sections present more risk than vaginal births in the psychological development of children (Hogle et al., 2003; Villar et al., 2007; Pasupathy and Smith, 2008; Wallin et al., 2010; Asztalos et al., 2016). Most of these studies relate birth by cesarean section to poorer neonatal outcomes, even after eliminating confounding factors related to the loss of fetal intrapartum well-being (Smith et al., 2004; Villar et al., 2007; Liston et al., 2008). Only in the case of breech presentations do cesareans provide greater protection compared to vaginal birth, both in single (Villar et al., 2007) and multiple pregnancies (Hogle et al., 2003). These unfavorable results after birth by cesarean section have been linked to onsets of respiratory distress and transient tachypnea secondary to a minor release of catecholamines by the fetus when it avoids the birth canal (Villar et al., 2007; Polidano et al., 2017), or even to defects in the expression of certain genes (UCP2) in the neurons of the fetal hippocampus in response to the absence of the physiological stress associated with vaginal delivery (Simón-Areces et al., 2012).

Several mechanisms could link the mode of delivery and child development. First, a cesarean section reduces the probability of breastfeeding and difficult maternal bonding, and induces changes in infant microbiomes, contributing to changes in the children’s metabolic pathways (Ajslev et al., 2011). Biochemical differences have been described in animal studies according to the route of birth. Dopamine concentrations in some areas of the prefrontal cortex, nucleus accumbens and striatum of rats and guinea pigs may be different depending on the mode of delivery, inducing differences in dopamine-mediated behaviors (El-Khodor and Boksa, 1997; Vaillancourt and Boksa, 2000). Additionally, amygdala and thalamus noradrenaline concentrations in adult rats may differ according to cesarean or vaginal birth (El-Khodor and Boksa, 2003). Although it is difficult to know the real implications of these differences in children, some studies show the influence of cesarean deliveries on an offspring’s microbiome. These studies indicate an alteration in the central nervous system that would affect short-term memory, motivation, mood and reactivity to stress, and raise questions about its long-term effects (Cryan and Dinan, 2012; Galland, 2014).

On the order hand, the rate of prematurity in the study sample is high in relation to the prevalence of prematurity in our environment. According to the data provided by the Andalusian Institute of Statistics (Institute of Statistics and Cartography of Andalusia, 2012), in our community, 5.9% of single pregnancies and 51.3% of twin pregnancies ended prematurely. The proportion of premature births in the sample is 67.7%, significantly higher than expected, which may be related to local clinical criteria, which reveal the existence of some bias in sample selection because the sample was made up of twin pregnancies that ended at a maximum level of complexity, which is an obstetric and perinatal center in the context of health-care in the province. However, we selected births from pregnancies that were more than 32 weeks, attempting to avoid cases in which extreme prematurity would directly influence the children’s cognitive development outcomes. The long-term effect of extreme prematurity is well documented (Wood et al., 2000). There is increased risk of cerebral palsy as well as problems in the development of basic or cognitive executive functions, conditioning low academic performance (Marlow et al., 2005, 2007; Wolke et al., 2008). The long-term effects of moderate (32–34 weeks) or late prematurity (34–37 weeks) are less obvious and, although there are some studies that reveal the existence of certain difficulties at school and in the cognitive development of infants born between 34 and 37 weeks (Lindstrom et al., 2007; Morse et al., 2009; Chyi et al., 2010), they have received less attention and there is less evidence of this (Odd et al., 2012). In the sample studied, 63% of the children born prematurely were born between 34 and 37 weeks, so the impact of prematurity on the outcomes of school performance, cognitive development, and intelligence is reduced. In addition, it is necessary to consider that any association between prematurity and psychological development may be due more to the causes of prematurity than to prematurity itself (Odd et al., 2012). To control the biases, we tried to control obstetric and prenatal variables, although it was not possible to obtain the effect of the type of delivery on the dependent variables analyzed, controlling the possible bias of the cesarean section, due to the number of cesarean sections found in the study sample. The relationship of this variable and other obstetric and perinatal variables (for example: antenatal drug/toxin exposure, congenital infection, and respiratory distress) with the psychological disorders of children would be relevant to analyze in larger samples in future studies. Likewise, factors in the initial design that could be related both to psychological development and prematurity have not been considered, as is the case, for example, with the mental health of parents and social class (Strenze, 2007; von Stumm et al., 2010; Odd et al., 2012; Polidano et al., 2017). These variables, however, could be analyzed together with prenatal and obstetric variables in later studies since, according to some studies, they are related with psychological development and intelligence. The lower the parental level of education and mental health, the lower the quality found in parental education practices, and in the development of the children (Strenze, 2007; von Stumm et al., 2010; Odd et al., 2012; Polidano et al., 2017).

On the other hand, although there is not a complete consensus in the literature (Ravi et al., 2018; Sacchi et al., 2018), we agree on the possible impact of intrauterine growth restriction (IUGR) on the long-term neurocognitive development of the children. The effects could be related to the hemodynamic and metabolic changes due to chronic fetal hypoxemia, fetal tissue hypoxia and acidosis that may occur during pregnancy (frequently due to placental insufficiency). The lack of information about the impact of IUGR on neurodevelopment can be considered one of the limitations of our study. In our initial design we included the assessment of the effects of fetal weight on neurodevelopment but, unfortunately, umbilical or cerebral arteries doppler velocimetry indexes were not available for most of the cases. As a short-term respiratory or metabolic outcome marker, we registered the scores of the Apgar test at the first and the fifth minute after birth, finding that although 8.9% of the children (11 cases) scored under 7 at first minute, the fifth minute score was fortunately normal in all cases, suggesting a good respiratory and metabolic state. The long-term effects of intrauterine growth restriction and specifically mild chronic hypoxemia could be considered in future studies.

The results confirm the importance of prenatal and perinatal variables, such as type of delivery, in psychological development, as shown by some studies (Calame et al., 2004; David and Dean, 2007; Lollar and Cordero, 2007; Cattani et al., 2010; Dall’oglio et al., 2010; Bidzan and Bieleninik, 2013; González-Valenzuela et al., 2015a; Asztalos et al., 2016). In addition, they are in line with those studies that establish negative effects of cesarean section on children’s microbiome and on psychological development (Hogle et al., 2003; Villar et al., 2007; Wallin et al., 2010; Cryan and Dinan, 2012; Galland, 2014; Asztalos et al., 2016; Polidano et al., 2017). However, these results should be considered with caution given the size of the sample, due to the difficulties encountered in assembling it. The creation of a provincial and national census on multiple births carried out by health institutions, as occurs in other countries (Bentley et al., 2016), would facilitate the availability of larger samples for study, as well as to carry out a follow-up and prevention of the possible psychological problems that this population may have. In future studies, it would be necessary to continue applying retrospective cohort designs to larger samples and at younger ages (2–3 years old) in order to generalize the results and ascertain whether the link between type of delivery and psychological development and intelligence is also manifested before compulsory schooling. It would also be interesting to carry out randomized prospective studies along the lines suggested by some authors (Barzilay et al., 2015), in order to find out if the difficulties in children’s psychological development diminish with time or if, on the contrary, they cause difficulties in school learning.

Finally, one of the implications of the results obtained could be that, in clinical practice, it might be advisable to carry out programs aimed at health professionals and parents to disseminate the risks of the type of delivery, in order to make known the effects of cesarean births, even in twins, with the idea of avoiding performing cesareans on demand or without medical indication. On the other hand, in light of the results obtained, another implication could be that detection protocols should be activated for possible infantile psychological difficulties in double births by cesarean at a very early age, through the neonatal and pediatric services within the health system.

## Author Contributions

EG-M, DL-M, and M-JG-V contributed conception and design of the study. DL-M, OC-G, and EG-M organized the database. DL-M performed the statistical analysis. DL-M, EG-M, and M-JG-V wrote sections of the manuscript. All authors wrote the first draft of the manuscript and contributed to manuscript revision, read and approved the submitted version.

## Conflict of Interest Statement

The authors declare that the research was conducted in the absence of any commercial or financial relationships that could be construed as a potential conflict of interest.

## Appendix

**Table A1:** Child Neuropsychological Maturity Questionnaire -CUMANIN- (Portellano et al., 2009).

Tests	Task/objective	Development area
*Articulation*	Repeating 15 words of increasing articulatory difficulty	VD
*Expressive language*	Repeating 4 sentences of increasing difficulty	
*Comprehensive language*	Answering 9 questions about the content of a story the child has heard	
*Psychomotricity*	Hopping on one leg Touching the nose with the finger Stimulation of the fingers Walking in balance Jumping with feet together Remain squatting with arms crossed Touching all the fingers of the hand	NVD
*Spatial structuring*	Performing 12 activities of spatial orientation through increasing difficulty Offered a psychomotor Graphomotor response	
*Visuoperception*	Reproducing 15 geometric drawings of increasing complexity	
*Iconic memory*	Memorizing 10 drawings of simple objects	
*Rhythm*	Reproducing 7 rhythmic series of increasing difficulty, through auditory presentation	
